# CHL Attenuates MIA-Induced Osteoarthritis by Regulating Inflammatory, Catabolic, and Anabolic Pathways Associated with Cartilage and Subchondral Bone Degeneration

**DOI:** 10.3390/nu18183031

**Published:** 2026-09-16

**Authors:** Sanjay, Mi-Seon Woo, Jihee Yoo, Tom Pfannenschmidt, Hae-Jeung Lee

**Affiliations:** 1 Department of Food and Nutrition, College of BioNano Technology, Gachon University, Seongnam 13120, Gyeonggi-do, Republic of Korea; sanjay.monga4@gmail.com or san55@gachon.ac.kr; 2 Institute for Aging and Clinical Nutrition Research, Gachon University, Seongnam 13120, Gyeonggi-do, Republic of Korea; 3 R&D Center, Chong Kun Dang Healthcare Corp., Yongsan-gu, Seoul 04300, Republic of Korea; mswoo@ckdhc.com; 4 CH Labs Corp., Yeongdeungpo-gu, Seoul 07249, Republic of Korea; jhyoo@chlabs.co.kr; 5 Innovation Management, K.-W. Pfannenschmidt GmbH, Habichthorst 36, 22459 Hamburg, Germany; drt@pfannenschmidt.de; 6 Department of Health Sciences and Technology, Gachon Advanced Institute for Health Science and Technology (GAIHST), Gachon University, Incheon 21999, Republic of Korea; 7 Gachon Biomedical Convergence Institute, Gachon University Gil Medical Center, Incheon 21565, Republic of Korea

**Keywords:** osteoarthritis, inflammation, cartilage degeneration, avocado/soybean unsaponifiables, CHL, extracellular matrix

## Abstract

**Background/Objectives**: Osteoarthritis (OA) is characterized by cartilage degeneration, extracellular matrix (ECM) disruption, inflammation, and impaired joint function. A standardized phytosterol-rich avocado/soybean unsaponifiable preparation (CHL) was examined in monosodium iodoacetate (MIA)-induced OA in Sprague–Dawley rats. **Methods**: Rats were orally administered low-, medium-, or high-dose CHL for 2 weeks before and 4 weeks after MIA induction. **Results**: CHL attenuated MIA-induced weight-bearing deficits and structural joint abnormalities. CHL also reduced cartilage degradation markers, including cartilage oligomeric matrix proteins and the C-terminal crosslinked telopeptide of type II collagen. CHL alleviated systemic inflammation by reducing serum interleukin-6, tumor necrosis factor-α, and prostaglandin E2 levels. Furthermore, CHL decreased the serum levels of matrix metalloproteinases (MMP)-1, MMP-2, MMP-9, and MMP-13, indicating reduced matrix-degrading responses. In joint cartilage tissues, CHL suppressed the mRNA expression of pro-inflammatory mediators and catabolic genes, such as *Mmp1*, *Mmp3*, and *Mmp9*. Additionally, CHL modulated cartilage anabolism and ECM-related markers by reducing collagen type I alpha 1 chain expression and enhancing SRY-box transcription factor 9, tissue inhibitor of metalloproteinase 1, and collagen type II alpha 1 chain expression. High-resolution liquid chromatography-mass spectrometry profiling of CHL revealed several tentatively identified compounds, such as (+)-discodermolide, epicoccamide, and brasilicardin C. **Conclusions**: Overall, these findings indicate that CHL may attenuate MIA-induced OA-associated functional impairment, structural alterations, inflammation, and cartilage matrix degradation, with medium doses showing the strongest protective effects. However, because CHL administration was initiated before OA induction, these findings primarily reflect preventive/protective effects and may not directly represent the therapeutic efficacy of CHL in established OA.

## 1. Introduction

Osteoarthritis (OA) is a leading cause of disability, affecting more than 607 million people worldwide as of 2021 and accounting for approximately 7.7% of the global population [1]. It substantially reduces the quality of life, particularly among aging populations, and is characterized by cartilage degradation, synovial inflammation, subchondral bone remodeling, pain, and impaired joint function [2]. Although OA has traditionally been considered a “wear-and-tear” disorder, it is now recognized as a multifactorial disease involving interconnected factors such as inflammation, oxidative stress (OS), extracellular matrix (ECM) degradation, and abnormal bone remodeling [3].

Among the various pathological mechanisms implicated in OA, inflammatory signaling is considered a major driver of disease progression and involves extensive crosstalk between the synovium and the articular cartilage [4]. Pro-inflammatory cytokines, including interleukin (IL)-1β, IL-6, and tumor necrosis factor (TNF)-α, initiate catabolic signaling cascades by activating nuclear factor kappa-light-chain-enhancer of activated B cells (NF-κB) and mitogen-activated protein kinase (MAPK) pathways. These responses can be further amplified by damage-associated molecular patterns generated in response to mechanical stress [5]. Activation of these pathways promotes the expression of cyclooxygenase-2 (COX-2) and inducible nitric oxide synthase (NOS2), resulting in an increased production of prostaglandin E2 (PGE_2_) and nitric oxide (NO). These inflammatory mediators further promote the polarization of synovial macrophages towards a proinflammatory M1 phenotype [6,7]. Sustained inflammatory signaling enhances the production of matrix metalloproteinases (MMPs), including MMP-1, MMP-3, and MMP-13, which degrade key extracellular matrix components such as type II collagen and aggrecan, ultimately contributing to the progressive loss of cartilage integrity [8].

The progressive destruction of articular cartilage is a hallmark of OA and is fundamentally driven by the disruption of ECM homeostasis, in which catabolic degradation substantially exceeds anabolic repair [9]. Persistent inflammation and increased activity of matrix-degrading enzymes accelerate the breakdown of ECM components, including type II collagen, aggrecan, and glycosaminoglycans (GAGs) [10]. These alterations are reflected by elevated levels of cartilage oligomeric matrix protein (COMP) and C-terminal cross-linked telopeptide of type II collagen (CTX-II), which serve as markers of cartilage matrix fragmentation and type II collagen degradation, respectively [11]. Furthermore, SRY-box transcription factor 9 (SOX9), a master regulator of chondrogenesis, is suppressed during cartilage degeneration, which results in reduced collagen type II alpha 1 (COL2A1) synthesis and impaired ECM regeneration [12]. Tissue inhibitors of metalloproteinases (TIMPs) also play a critical role in maintaining the structural and functional integrity of cartilage by regulating MMP activity [13]. However, mitochondrial dysfunction in chondrocytes increases the generation of reactive oxygen species (ROS), which further exacerbates matrix degradation by depleting TIMPs, while simultaneously promoting excessive MMP activity [13]. Collectively, these interconnected processes establish a self-perpetuating cycle involving inflammation, OS, and ECM destruction, thereby driving progressive cartilage deterioration in patients with OA.

The multifactorial nature of OA presents significant challenges for effective treatment. Although nonsteroidal anti-inflammatory drugs (NSAIDs) and glucocorticoids are widely available, their use is associated with adverse health effects, including gastric ulcers [14]. Moreover, these treatments primarily provide symptomatic pain relief and do not directly reverse the underlying pathological changes associated with OA [15]. Therefore, there is an increasing need to identify safer and more effective therapeutic options, particularly natural compounds with limited adverse effects and multi-target activities. These compounds may offer a more comprehensive therapeutic approach than conventional treatments that primarily focus on pain management.

Avocado and soybean unsaponifiables (ASU) are naturally derived lipid extracts obtained from the unsaponifiable fractions of *Persea americana* and *Glycine max*, respectively, and are enriched in phytosterols, tocopherols, and other minor lipophilic constituents [16,17]. Phytosterols have gained considerable attention because of their anti-inflammatory, antioxidant, and cartilage-protective properties [18,19]. Previous studies have shown that ASU suppresses inflammatory cytokine production, inhibits MMP expression, and promotes ECM synthesis by chondrocytes, thereby contributing to the maintenance of cartilage homeostasis [20,21,22]. Clinical studies have also reported improvements in pain and joint function following ASU supplementation in patients with OA, supporting its potential as a disease-modifying nutraceutical [23,24,25,26]. Importantly, ASU preparations are not chemically identical, and their compositions may vary depending on the source materials, manufacturing processes, and degree of standardization. A comparative analysis of seven commercially available ASU products demonstrated substantial differences in their unsaponifiable composition, including sterol and tocopherol content and other lipid fractions, as well as differences in their pharmacological effects on human osteoarthritic chondrocytes [27]. These findings indicate that the biological effects observed with one ASU preparation are not necessarily present in other ASU formulations.

Although ASU have previously been investigated in experimental OA, including monosodium iodoacetate (MIA)-induced OA, studies have evaluated different ASU preparations using a range of structural, inflammatory, and pain-related outcomes [28,29]. However, the biological effects of a specific standardized ASU preparation should be evaluated independently from those of previously investigated ASU formulations. CHL (Avocadoflex^®^) is a specific standardized ASU preparation for which limited information is available regarding its effects when administered before and throughout the development of MIA-induced OA.

MIA-induced OA model reproduces several pathological and behavioral characteristics of human OA, including cartilage destruction, inflammatory responses, subchondral bone alterations, and pain-associated functional impairment [30,31,32]. Therefore, this model is useful for evaluating therapeutic candidates that target the inflammatory and degenerative responses associated with OA. In this study, we investigated the effects of CHL, a standardized phytosterol-rich formulation, in an MIA-induced OA rat model. We evaluated CHL across multiple levels of OA-related pathology, including weight-bearing function, joint structural changes, cartilage degeneration, inflammatory biomarkers, serum MMPs, and inflammatory, catabolic, anabolic, and ECM-related gene expression in the joint cartilage. High-resolution liquid chromatography-mass spectrometry (HR-LC/MS) profiling was performed to characterize the chemical profile of CHL. This integrated approach was designed to provide a broader characterization of the effects of CHL on MIA-induced OA.

## 2. Materials and Methods

### 2.1. Materials

The test material used in the present study was CHL, the designation used herein for the specific commercial avocado/soybean unsaponifiable preparation (ASU; commercial name: Avocadoflex^®^) obtained from K.-W. Pfannenschmidt GmbH (Hamburg, Germany, GmbH). The preparation consists of unsaponifiable fractions derived from *Persea americana* fruit and *Glycine max* seeds and is specified by the manufacturer to contain a minimum of 30% total phytosterols, determined as the sum of β-sitosterol, campesterol, and stigmasterol. The batches used in this study complied with the manufacturer’s instructions (version 1.6).

The MIA and corn oil were purchased from Sigma-Aldrich (St. Louis, MO, USA). Hana Pharm Co., Ltd. (Gangnam, Republic of Korea) provided isoflurane. Rat Enzyme-linked immunosorbent assay (ELISA) kits for COMP, CTX-II, PGE_2_, TNF-α, IL-10, IL-1β, IL-12, IL-6, and MMPs (MMP-1, -2, -3, -9, and -13) were purchased from ElabScience (Wuhan, Hubei, China). ELISA kits for GAGs and aggrecan were obtained from Biomatik (Kitchener, ON, Canada).

### 2.2. High-Resolution Liquid Chromatography-Mass Spectrometry (HR-LC/MS) Analysis of CHL

The CHL was completely dissolved prior to HR-LC/MS analysis. Thermo Scientific Orbitrap Exploris 120 mass spectrometer (San Jose, CA, USA) equipped with a Hypersil GOLD™ C18 (Waltham, MA, USA) column was used for HR-LC/MS analysis. The column was maintained at a temperature of 40 °C with a flow rate of 0.2 mL/min for chromatographic separation under isocratic conditions. A negative ionization mode was used to acquire the mass spectra using a heated electrospray ionization (H-ESI) source over an *m*/*z* range of 100–1500. The total ion chromatogram (TIC) obtained from the CHL was compared with that of a blank control to distinguish sample-associated signals from background or solvent-related signals. Detected compounds were tentatively annotated by comparison of the acquired high-resolution mass spectral data with reference spectra in the mzCloud and mzVault libraries using negative-ion ([M−H]^−^) mode [33,34].

### 2.3. Experimental Animals

Forty-eight 4-week-old Sprague–Dawley (SD) rats weighing approximately 170–200 g were procured from Raon Bio (Siheung, Gyeonggi-do, Republic of Korea). The animals were acclimatized for 1 week in a conventional animal care facility under controlled environmental conditions, with a temperature of 22 ± 2 °C, relative humidity of 50 ± 10%, and a 12 h light (200–300 lx)/12 h dark cycle. The rats had ad libitum access to sterilized drinking water and a standard pellet diet (ENVIGO, Indianapolis, IN, USA). All experimental procedures involving animals were approved by the Institutional Animal Care and Use Committee (IACUC) of Daejeon University (approval number: DJUARB2025-014; approved on 13 May 2025) and were conducted in accordance with the ethical guidelines for animal experimentation.

After a 1-week acclimatization period, the animals were weighed and randomly divided into six groups such that the mean body weights were comparable among the different groups (*n* = 8/group): normal control (NC), OA-induced control (MIA), MIA + celecoxib (positive control, PC), and three CHL treatment groups receiving low (LOW), medium (MID), and high (HIGH) doses. The PC group was orally administered celecoxib at 10 mg/kg/day, whereas the LOW, MID, and HIGH groups received CHL at doses of 10, 30, and 300 mg/kg/day, respectively, for 6 weeks. After 2 weeks of pre-administration, OA was induced in all groups except for the NC group. As CHL is not water-soluble, it was uniformly dissolved in corn oil prior to oral administration. Equivalent volumes of corn oil were administered to the NC and MIA groups throughout the experimental period.

**Figure 1:**
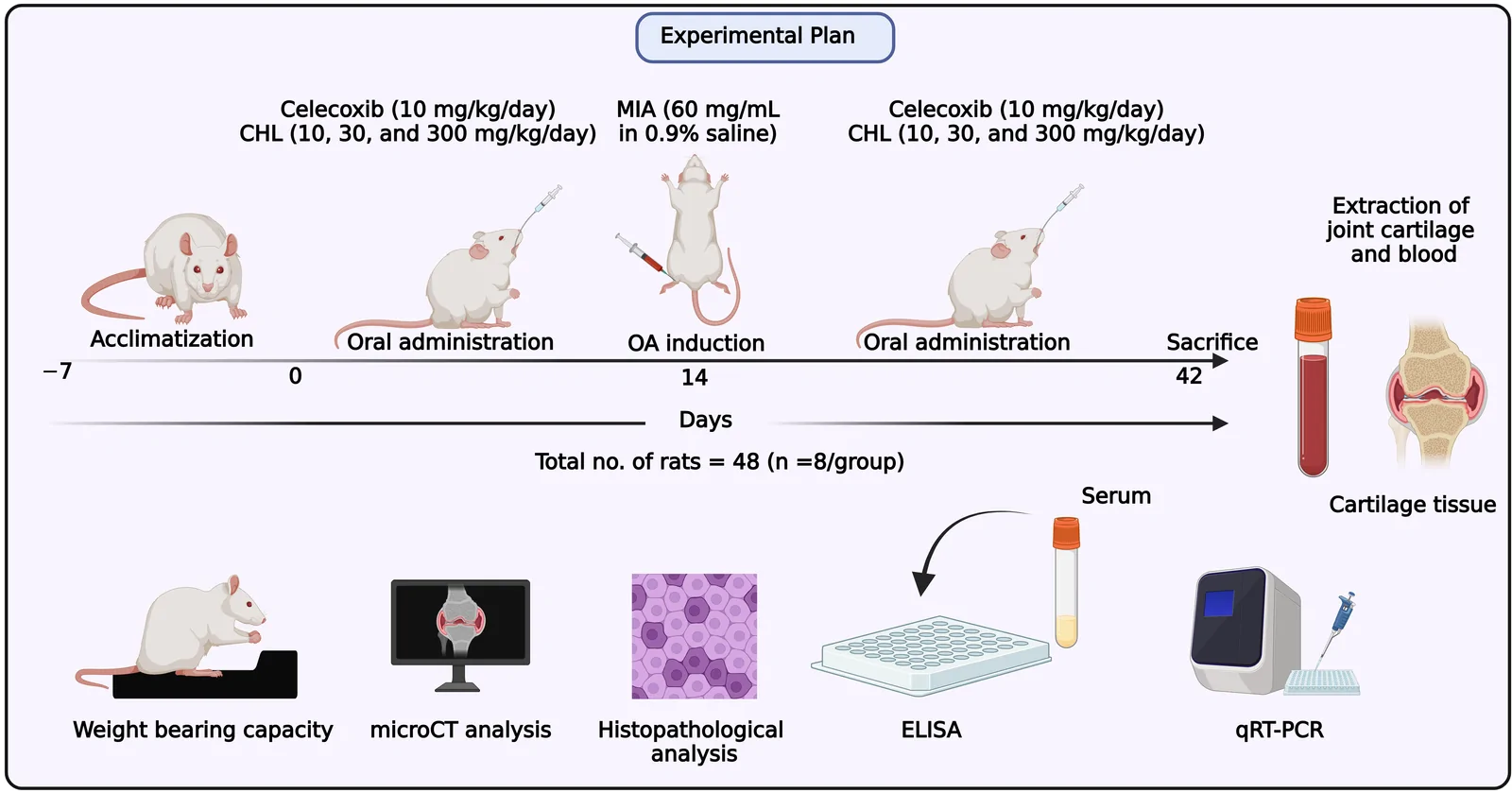
Experimental setup. SD rats were randomly divided into six groups (*n* = 8/group): NC, MIA, MIA + celecoxib (PC, 10 mg/kg/day), and CHL-treated LOW (10 mg/kg/day), MID (30 mg/kg/day), and HIGH (300 mg/kg/day) groups. CHL and celecoxib were administered orally for 6 weeks, with OA induced after 2 weeks of pre-administration by intra-articular injection of MIA. Weight-bearing capacity assessment, micro-CT analysis of the joints, H&E staining, ELISA-based measurement of osteoarthritis-related serum biomarkers, and cartilage tissue gene expression analysis associated with cartilage degeneration were subsequently performed. CHL, standardized phytosterol-rich avocado/soybean unsaponifiable preparation; ELISA, enzyme-linked immunosorbent assay; H&E, hematoxylin and eosin; MIA, monosodium iodoacetate; micro-CT, micro-computed tomography; NC, normal control; OA, osteoarthritis; PC, positive control (celecoxib); SD, Sprague–Dawley.

For OA induction, animals were anesthetized with 3% isoflurane. Following shaving of the right knee joint area, 50 μL of MIA solution (60 mg/mL in 0.9% saline; Sigma-Aldrich, St. Louis, MO, USA) was administered intra-articularly into the right knee joint under sterile conditions. A detailed overview of the experimental design is presented in Figure 1.

### 2.4. Weight-Bearing Measurements

To evaluate the pain and functional impairment associated with OA, hind limb weight-bearing capacity in SD rats was assessed at the 2nd and 4th week after OA induction using an incapacitance test meter (IITC Life Science, Woodland Hills, CA, USA). The animals were placed in a plastic holder with both hind paws positioned on force plates. The weight distribution of each hind limb was continuously recorded for 30 s, and the average value was calculated. The weight-bearing distribution on the injured right hind limb was expressed as a percentage using the following equation:

$$\mathrm{Weight}\text{-}\mathrm{bearing}\;\mathrm{capacity}\;\%\;(right\;limb)=\frac{Weight\;in\;the\;right\;hind\;limb}{\left(Weight\;in\;the\;right\;hind\;limb+Weight\;in\;the\;left\;hind\;limb\right)}$$

### 2.5. Enzyme-Linked Immunosorbent Assay (ELISA)

At the end of the experimental period, blood samples were collected via cardiac puncture under inhalation anesthesia. The collected blood was allowed to clot at room temperature for approximately 30 min and then centrifuged at 3000 rpm for 15 min to separate the serum. The isolated serum was immediately aliquoted (200 μL per tube) and stored at −80 °C until analysis. Before analysis, the samples were thawed and processed.

Quantitative analysis of serum biomarkers associated with cartilage degradation and inflammation was performed using ELISA. Commercial rat-specific ELISA kits were obtained from Biomatik (Kitchener, ON, Canada) and Elabscience (Wuhan, China), and all assays were performed according to the manufacturer’s instructions. Briefly, standards and serum samples were added to precoated 96-well plates and incubated with primary and enzyme-conjugated antibodies. After repeated washing with buffer to remove nonspecific binding, the TMB substrate solution was added for color development. The reaction was terminated with a stop solution, and absorbance was measured at 450 nm using a microplate reader (Molecular Devices, San Jose, CA, USA). The concentration of each sample was calculated using the corresponding standard curve.

### 2.6. Real-Time Quantitative PCR (qRT-PCR)

At the end of the experimental period, the animals were sacrificed, and knee joint cartilage tissues were collected. Total RNA was extracted from the tissues using a total RNA prep kit (Intronbio, Seongnam, Republic of Korea). Briefly, 50 ng of RNA was reverse-transcribed into complementary DNA using AccuPower CycleScript RT premix (Bioneer, Daejeon, Republic of Korea). Reverse transcription was performed using a PCR cycler (PCRmax, Cheshire, UK) at 45 °C for 60 min, followed by 95 °C for 5 min. The synthesized cDNA was used for real-time PCR analysis. The reaction mixture was prepared in a total volume of 20 μL, consisting of 10 μL of qPCRBIO SyGreen mix (PCR Biosystems, London, UK), 5.5 μL of synthesized cDNA, 2.5 μL of DEPC, and 1 μL of each of the forward and reverse primers. Amplification was performed under the following conditions: initial denaturation at 95 °C for 2 min, followed by 40 cycles of 95 °C for 5 s and 62.5 °C for 30 s. Relative gene expression levels were calculated using the comparative 2^−ΔΔCt^ method and normalized to β-actin expression, with the MIA group used as the calibrator. Detailed primer sequences are listed in Table 1.

### 2.7. micro-CT Analysis of Knee Tissue

At the end of the experimental period, the right knee joints were collected from sacrificed animals for morphological analysis using micro-computed tomography (micro-CT). Harvested knee joints were carefully removed from the surrounding soft tissue and fixed in 4% paraformaldehyde (Sigma-Aldrich) for 24 h. The samples were subsequently used for scanning through a Quantum FX micro-CT system (PerkinElmer, Shelton, CT, USA), maintained at a tube voltage of 90 kVp, tube current of 160 μA, and acquisition time of 120 s, with a field of view (FOV) of 20 mm and a pixel size of 40 μm. The obtained raw image data were reconstructed and analyzed using Analyze 12 software (Mayo Clinic, Rochester, MN, USA). For image noise reduction, a median filter (3 × 3 × 3) was applied, followed by grayscale-based masking using a 4000–9000 threshold range. An Object Separator was used to separate individual menisci and generate mask maps. The total meniscal volume (mm^3^) was quantitatively calculated from the resulting segmented mask. The segmented images were used for three-dimensional volume rendering to obtain a 3D reconstruction of the menisci.

### 2.8. Histopathological Analysis of Knee Tissue

Following completion of the experiment, knee joint tissues were excised and immediately fixed in 4% paraformaldehyde solution for 48 h. The tissues were subsequently decalcified in a 10% EDTA solution for 3 weeks. Following decalcification, the samples were washed under running water and embedded in paraffin to prepare blocks. The blocks were sectioned into 5 μm-thick sections using a microtome (Leica, Wetzlar, Germany) and mounted onto glass slides. Hematoxylin and eosin (H&E) staining was performed. The stained sections were imaged using a digital slide scanner (Motic, Xiamen, China), and histopathological alterations were evaluated.

### 2.9. Statistical Analysis

The results are presented as the mean ± standard deviation (SD). All the experiments were performed using biological replicates obtained from different animals. Each experimental animal was considered an independent biological replicate, and no technical replicates were performed. Statistical analyses were performed using GraphPad Prism 10.0.0 (GraphPad Software, San Diego, CA, USA). One-way analysis of variance (ANOVA) followed by Tukey’s post-hoc multiple comparison test was used for single-endpoint analyses. Two-way repeated-measures ANOVA was used to analyze repeated measurements over time. A *p*-value < 0.05 was considered statistically significant.

## 3. Results

### 3.1. HR-LC/MS Characterization of the Tested Compound

**Figure 2:**
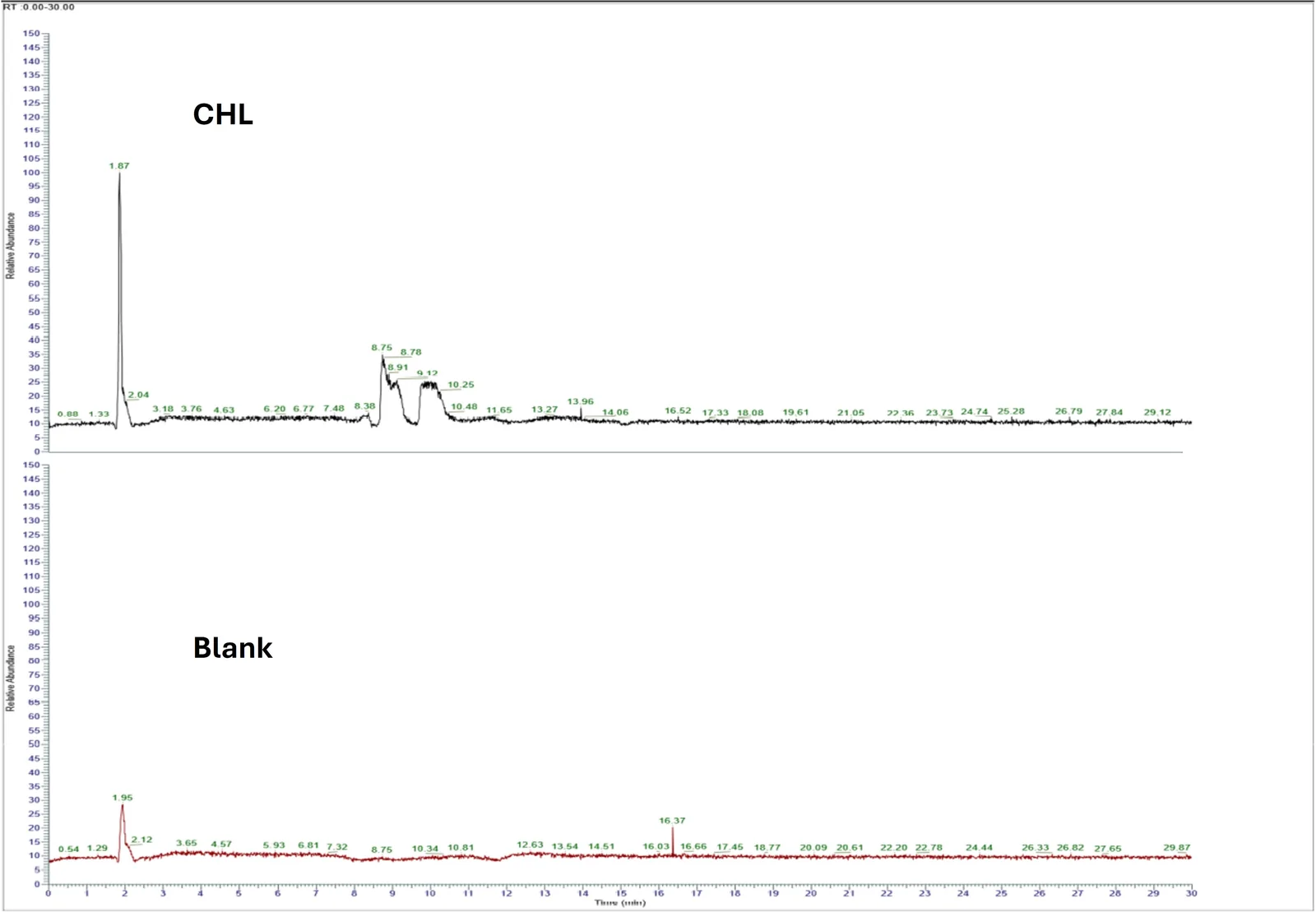
High-Resolution Liquid Chromatography-Mass Spectrometry (HR-LC/MS) total ion chromatograms (TICs) acquired in negative ionization mode.

Chromatographic characterization of CHL in negative ionization mode revealed several distinct peaks compared to the blank control. Spectral library searching tentatively revealed ten primary components eluting across three main retention time regions, including early-eluting unknown peaks (RT 1.87–1.94 min and 8.38 min), a dense middle cluster of secondary metabolites and organic compounds (RT 8.75–9.11 min), and late-eluting derivatives (RT 9.98–10.01 min). Among the library-matched compounds, (+)-discodermolide, epicoccamide, and brasilicardin C were the predominant components based on the relative peak area (Figure 2). A detailed summary of the detected peaks, including putative compounds, retention times, peak areas, CAS numbers, and molecular weights, is presented in Table 2. Because the library match scores did not reach the high-confidence threshold (BM<80.0), the match scores were lower; therefore, the compound assignments were considered tentative and require further confirmation using authentic chemical standards. Further confirmation using authentic reference standards and/or additional structural characterizations, including MS/MS analysis, is required to establish the identities of these compounds.

### 3.2. CHL Improves Weight-Bearing Capacity and Attenuates Structural Deformities in MIA-Induced OA Rats

**Figure 3:**
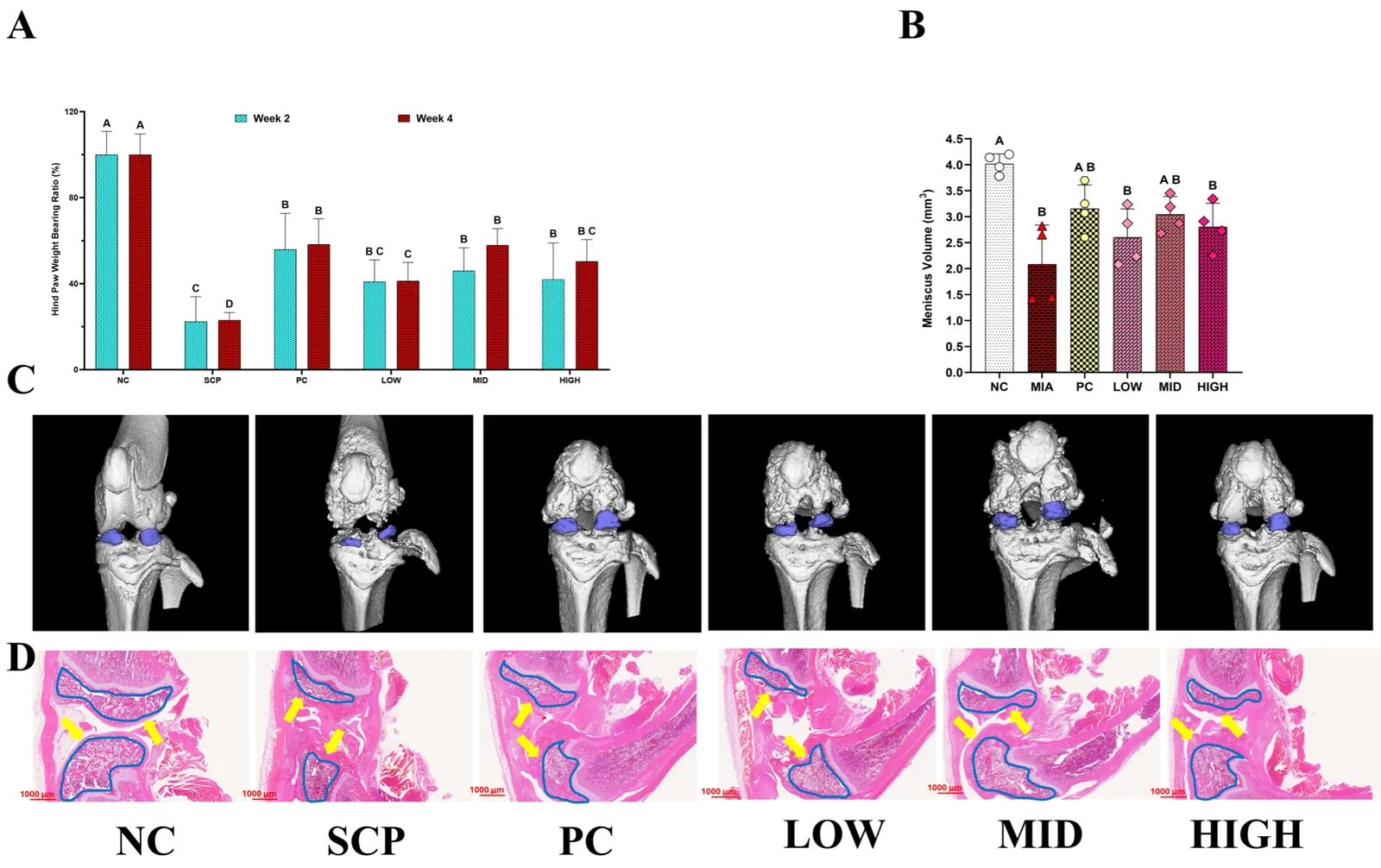
Effects of CHL on hind-paw weight-bearing ratio and structural deformities in MIA-induced osteoarthritic rats. SD rats were randomly divided into six groups (*n* = 8/group): NC, MIA, MIA + celecoxib (PC, 10 mg/kg/day), and CHL-treated LOW (10 mg/kg/day), MID (30 mg/kg/day), and HIGH (300 mg/kg/day) groups. CHL and celecoxib were administered orally for 6 weeks, with OA induced after 2 weeks of pre-administration by intra-articular injection of MIA. (**A**) Weight-bearing distribution was evaluated using an incapacitance tester at weeks 2 and 4 after MIA injection. The weight-bearing ratio was calculated as follows: Weight-bearing capacity % (right limb) = $\frac{Weight\;in\;the\;right\;hind\;limb}{\left(Weight\;in\;the\;right\;hind\;limb+Weight\;in\;the\;left\;hind\;limb\right)}$. (**B**,**C**) Micro-CT analysis of the experimental limb was performed, and meniscus volume was measured. (**D**) H&E staining of experimental limbs was performed. Values are presented as the mean ± SD (*n* = 8). Statistical significance is indicated by distinct letters, with different letters indicating statistically significant differences between groups.

The weight-bearing capacity is an important indicator of pain severity in OA-treated animals. The hind-paw weight-bearing ratio was assessed at the 2nd and 4th weeks after MIA injection. The MIA-induced group exhibited a significant reduction in the weight-bearing ratio; however, oral administration of PC and all three doses of CHL (LOW, MID, and HIGH) significantly reversed this MIA-induced reduction, as demonstrated by the increased weight-bearing ratios compared with the MIA-induced group (Figure 3A). Micro-CT analysis was performed to observe the effects of CHL on MIA-induced joint structural changes in SD rats. The presence of irregular articular surfaces and subchondral bone alterations was observed visually in MIA-induced rats, which decreased in the PC- and CHL-treated groups. In addition, quantitative analysis of meniscal volume showed a significant reduction in MIA-induced rats, which tended to be restored in the PC- and CHL-treated groups, as the PC- and CHL-treated groups showed a non-significant increase in meniscal volume (Figure 3B,C). Furthermore, H&E staining revealed synovial tissue loss and structural deformation in MIA-induced rats, whereas these alterations were reduced in the PC- and CHL-treated groups (Figure 3D). The histopathological findings were evaluated qualitatively based on the observed tissue morphology, and no validated histopathological scoring system was applied.

### 3.3. CHL Regulates Serum Biomarkers Associated with Cartilage Degradation and ECM Metabolism Deformities in MIA-Induced OA Rats

**Figure 4:**
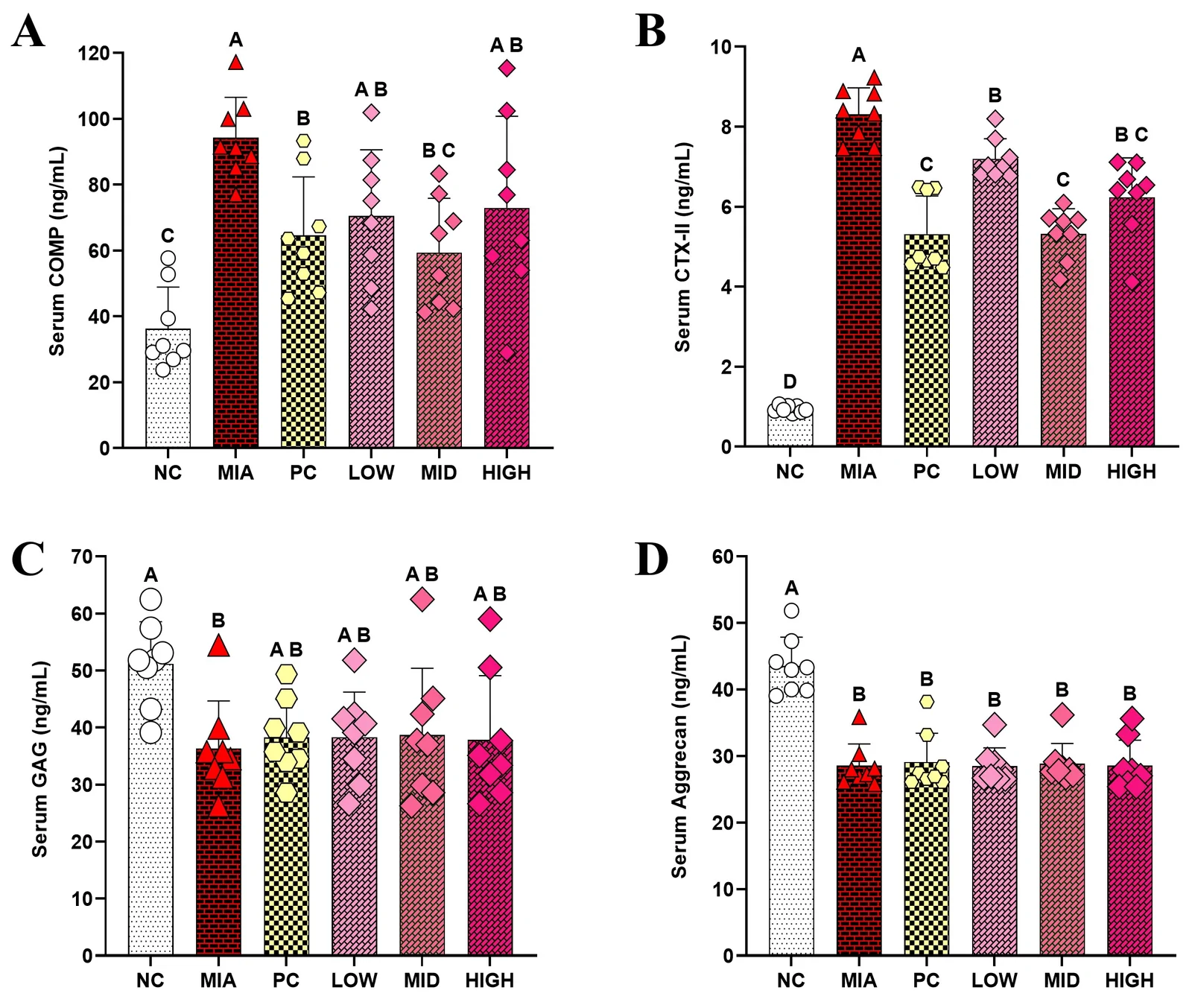
Effects of CHL on serum biomarkers associated with cartilage degradation and ECM metabolism. SD rats were randomly divided into six groups (*n* = 8/group): NC, MIA, MIA + celecoxib (PC, 10 mg/kg/day), and CHL-treated LOW (10 mg/kg/day), MID (30 mg/kg/day), and HIGH (300 mg/kg/day) groups. CHL and celecoxib were administered orally for 6 weeks, with OA induced after 2 weeks of pre-administration by intra-articular injection of MIA. Serum levels of (**A**) COMP, (**B**) CTX-II, (**C**) GAG and (**D**) aggrecan were measured using ELISA kits. Values are presented as the mean ± SD (*n* = 8). Statistical significance is indicated by distinct letters, with different letters indicating statistically significant differences between groups. COMP, cartilage oligomeric matrix protein; CTX-II, C-terminal crosslinked telopeptide of type II collagen; and GAG, glycosaminoglycans.

To evaluate the effects of CHL on serum biomarkers associated with cartilage degradation and ECM metabolism, cartilage-related biomarkers including COMP, CTX-II, aggrecan, and GAG were measured in all experimental groups. MIA-induced SD rats exhibited increased serum levels of COMP and CTX-II, accompanied by decreased levels of GAG and aggrecan, consistent with increased cartilage degradation and altered ECM-related biomarkers. Oral administration of PC and CHL, particularly at the MID-dose, significantly reduced the elevated serum levels of COMP and CTX-II, except in the HIGH-dose group. However, neither PC nor CHL treatment significantly affected the MIA-induced reduction in serum GAG and aggrecan levels (Figure 4A–D).

### 3.4. CHL Modulates Serum Inflammatory Mediators in MIA-Induced OA Rats

**Figure 5:**
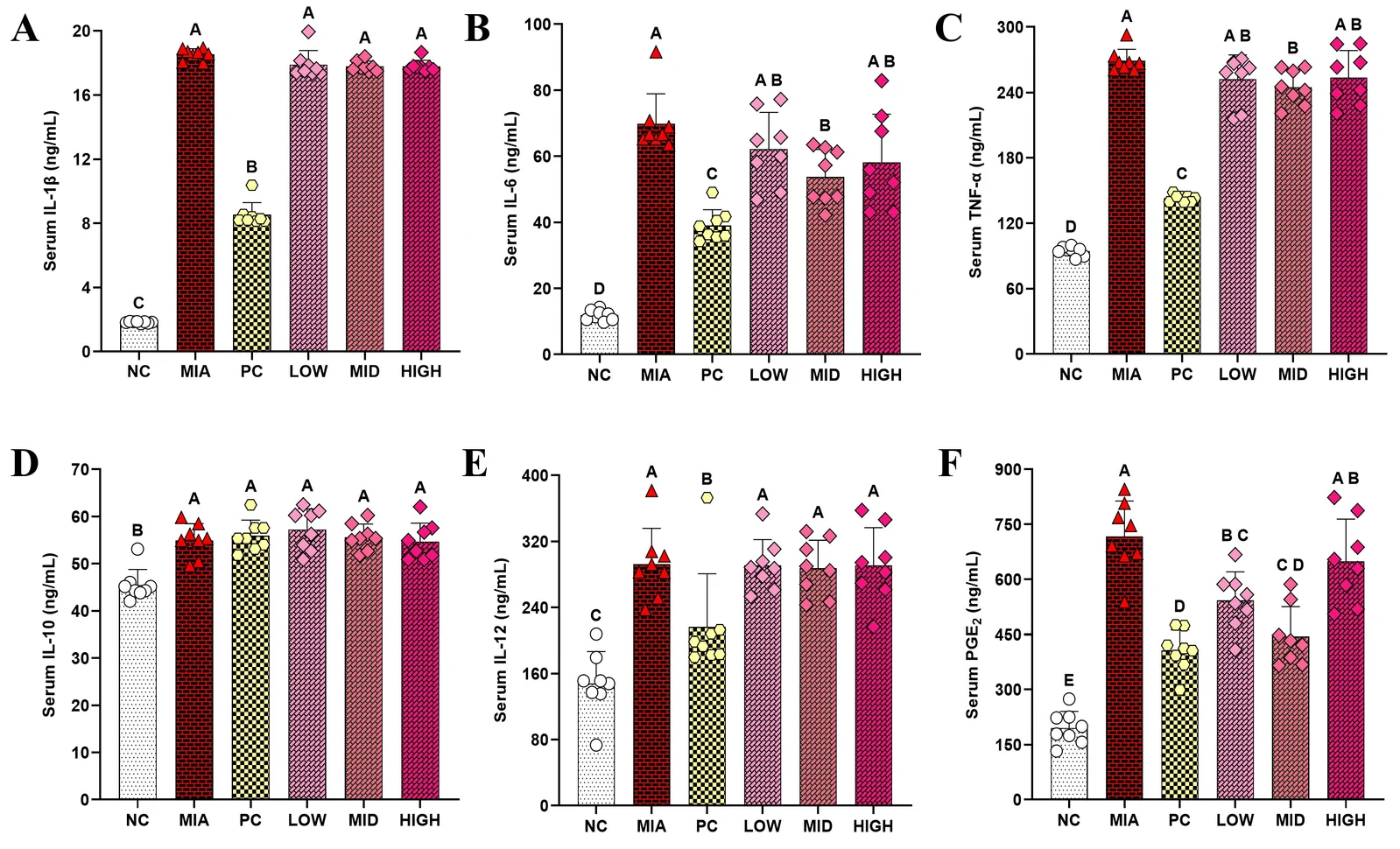
Effects of CHL on serum biomarkers associated with cartilage degradation and ECM metabolism. SD rats were randomly divided into six groups (*n* = 8/group): NC, MIA, MIA + celecoxib (PC, 10 mg/kg/day), and CHL-treated LOW (10 mg/kg/day), MID (30 mg/kg/day), and HIGH (300 mg/kg/day) groups. CHL and celecoxib were administered orally for 6 weeks, with OA induced after 2 weeks of pre-administration by intra-articular injection of MIA. Serum levels of (**A**) IL-1β, (**B**) IL-6, (**C**) TNF-α, (**D**) IL-10, (**E**) IL-12, and (**F**) PGE_2_ were measured using ELISA kits. Values are presented as the mean ± SD (*n* = 8). Statistical significance is indicated by distinct letters, with different letters indicating statistically significant differences between groups. IL, interleukin; TNF-α, tumor necrosis factor-α; and PGE_2_, prostaglandin E2.

To evaluate the effects of CHL on systemic inflammatory biomarkers in MIA-induced OA rats, serum levels of the pro-inflammatory mediators IL-1β, IL-6, TNF-α, IL-12, and PGE_2_, as well as the anti-inflammatory cytokine IL-10, were measured. MIA induction markedly increased the serum levels of these inflammatory mediators compared with those in the NC group. Although CHL treatment did not consistently suppress all MIA-induced inflammatory markers, the MID-dose group showed significant reductions in serum IL-6 and TNF-α levels. In addition, serum PGE_2_ levels were significantly decreased in the LOW- and MID-dose groups (Figure 5A–F).

### 3.5. CHL Attenuates Serum Matrix Metalloproteinases in MIA-Induced OA Rats

**Figure 6:**
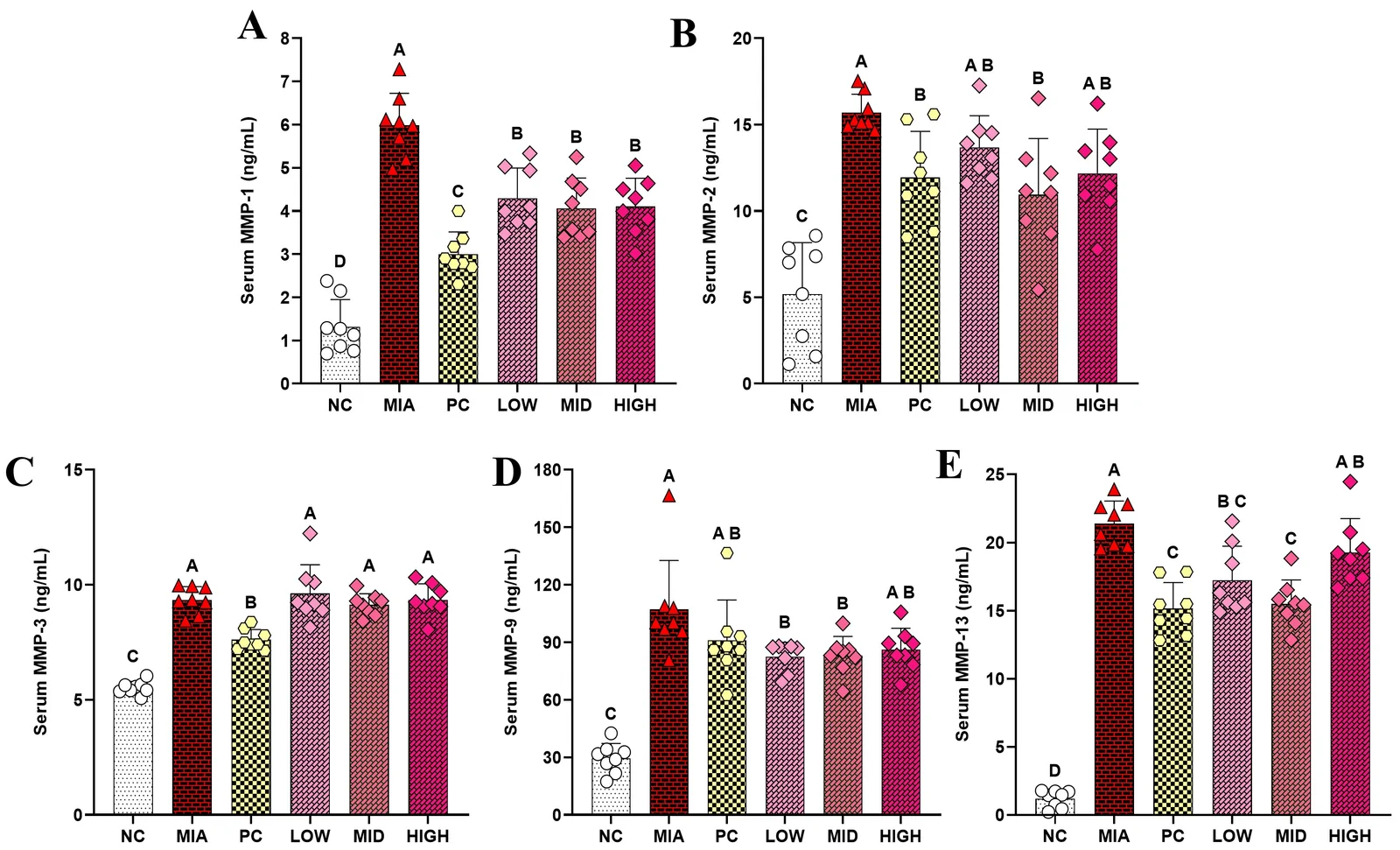
Effects of CHL on serum matrix metalloproteinases in MIA-induced OA rats. SD rats were randomly divided into six groups (*n* = 8/group): NC, MIA, MIA + celecoxib (PC, 10 mg/kg/day), and CHL-treated LOW (10 mg/kg/day), MID (30 mg/kg/day), and HIGH (300 mg/kg/day) groups. CHL and celecoxib were administered orally for 6 weeks, with OA induced after 2 weeks of pre-administration by intra-articular injection of MIA. Serum levels of (**A**) MMP-1, (**B**) MMP-2, (**C**) MMP-3, (**D**) MMP-9, and (**E**) MMP-13 were observed using ELISA kits. Values are presented as the mean ± SD (*n* = 8). Statistical significance is indicated by distinct letters, with different letters indicating statistically significant differences between groups. MMPs, matrix metalloproteinases.

To investigate the effects of CHL on serum MMP levels in MIA-induced OA rats, serum levels of MMP-1, MMP-2, MMP-3, MMP-9, and MMP-13 were evaluated across all experimental groups. MIA induction significantly increased the serum levels of all the assessed MMPs, consistent with the increased expression of matrix-degrading enzymes. However, oral administration of CHL, particularly at the MID-dose, significantly reduced the serum levels of MMP-1, MMP-2, MMP-9, and MMP-13. The PC group showed effects comparable to those observed in the MID group (Figure 6A–E).

### 3.6. CHL Modulates Inflammatory and Catabolic Gene Expression in Joint Cartilage of OA Rats

**Figure 7:**
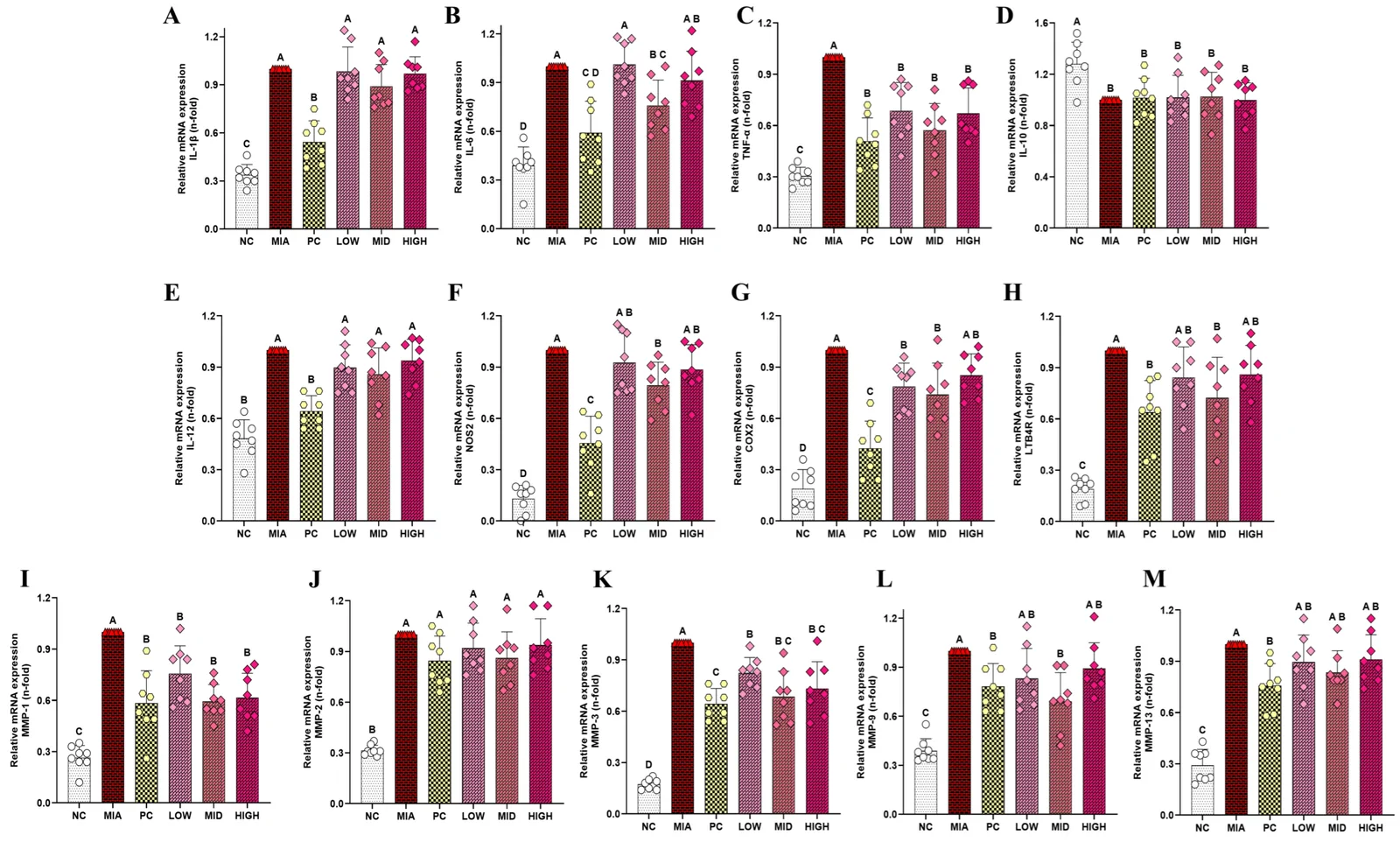
Effects of CHL on inflammatory and catabolic gene expression in joint cartilage of OA rats. SD rats were randomly divided into six groups (*n* = 8/group): NC, MIA, MIA + celecoxib (PC, 10 mg/kg/day), and CHL-treated LOW (10 mg/kg/day), MID (30 mg/kg/day), and HIGH (300 mg/kg/day) groups. CHL and celecoxib were administered orally for 6 weeks, with OA induced after 2 weeks of pre-administration by intra-articular injection of MIA. The mRNA expression levels of (**A**–**H**) inflammatory mediators, including *Il1b*, *Il6*, *Tnfa*, *Il10*, *Il12*, *Nos2*, *Cox2*, and *Ltb4r*, and (**I**–**M**) Mmps, including *Mmp1*, *Mmp2*, *Mmp3*, *Mmp9*, and *Mmp13*, were measured in joint cartilage tissue of OA rats using qRT-PCR. Values are presented as the mean ± SD (*n* = 8). Statistical significance is indicated by distinct letters, with different letters indicating statistically significant differences between groups. IL, interleukin; *Tnfa*, tumor necrosis factor-alpha; *Nos2*, nitric oxide synthase 2; *Cox2*, cyclooxygenase 2; *Ltb4r*, leukotriene B4 receptor; and Mmps, matrix metalloproteinases.

To further investigate the effects of CHL on inflammatory and catabolic gene expression in joint cartilage of OA rats, the mRNA expression levels of inflammatory mediators, including *Il1b*, *Il6*, *Tnfa*, *Il12*, *Nos2*, *Cox2*, and *Ltb4r*, as well as the anti-inflammatory cytokine *Il10* and matrix-degrading enzymes, including *Mmp1*, *Mmp2*, *Mmp3*, *Mmp9*, and *Mmp13*, were evaluated across all experimental groups. The mRNA expression levels of the pro-inflammatory mediators *Il1b*, *Il6*, *Tnfa*, *Il12*, *Nos2*, *Cox2*, and *Ltb4r* were significantly elevated in the joint cartilage tissues of MIA-induced OA rats. However, oral administration of CHL, particularly at the MID dose, significantly reduced the expression levels of *Il6*, *Tnfa*, *Nos2*, *Cox2*, and *Ltb4r*, while having no significant effect on *Il1b* and *Il12* expression. The PC group also showed significant attenuation of MIA-induced inflammatory mediator expression (Figure 7A–H). In contrast, mRNA expression of the anti-inflammatory cytokine *Il10* was significantly downregulated in MIA-induced OA rats, whereas neither PC nor CHL treatment at any tested dose significantly restored *Il10* expression (Figure 7D).

Furthermore, MIA induction significantly increased the mRNA expression levels of *Mmp1*, *Mmp2*, *Mmp3*, *Mmp9*, and *Mmp13* in the joint cartilage tissues, consistent with the increased expression of matrix-degrading enzymes. Treatment with CHL, particularly at the MID-dose, significantly reduced the mRNA expression levels of *Mmp1*, *Mmp3*, and *Mmp9*. The PC group also showed a significant reduction in the expression of *Mmp1*, *Mmp3*, *Mmp9*, and *Mmp13* in joint cartilage tissues of MIA-induced OA rats. Neither PC nor CHL treatment significantly affected *Mmp2* mRNA expression in joint cartilage tissues (Figure 7I–M).

### 3.7. CHL Modulates Cartilage Anabolic Markers and ECM-Related Gene Expression in Joint Cartilage of OA Rats

**Figure 8:**
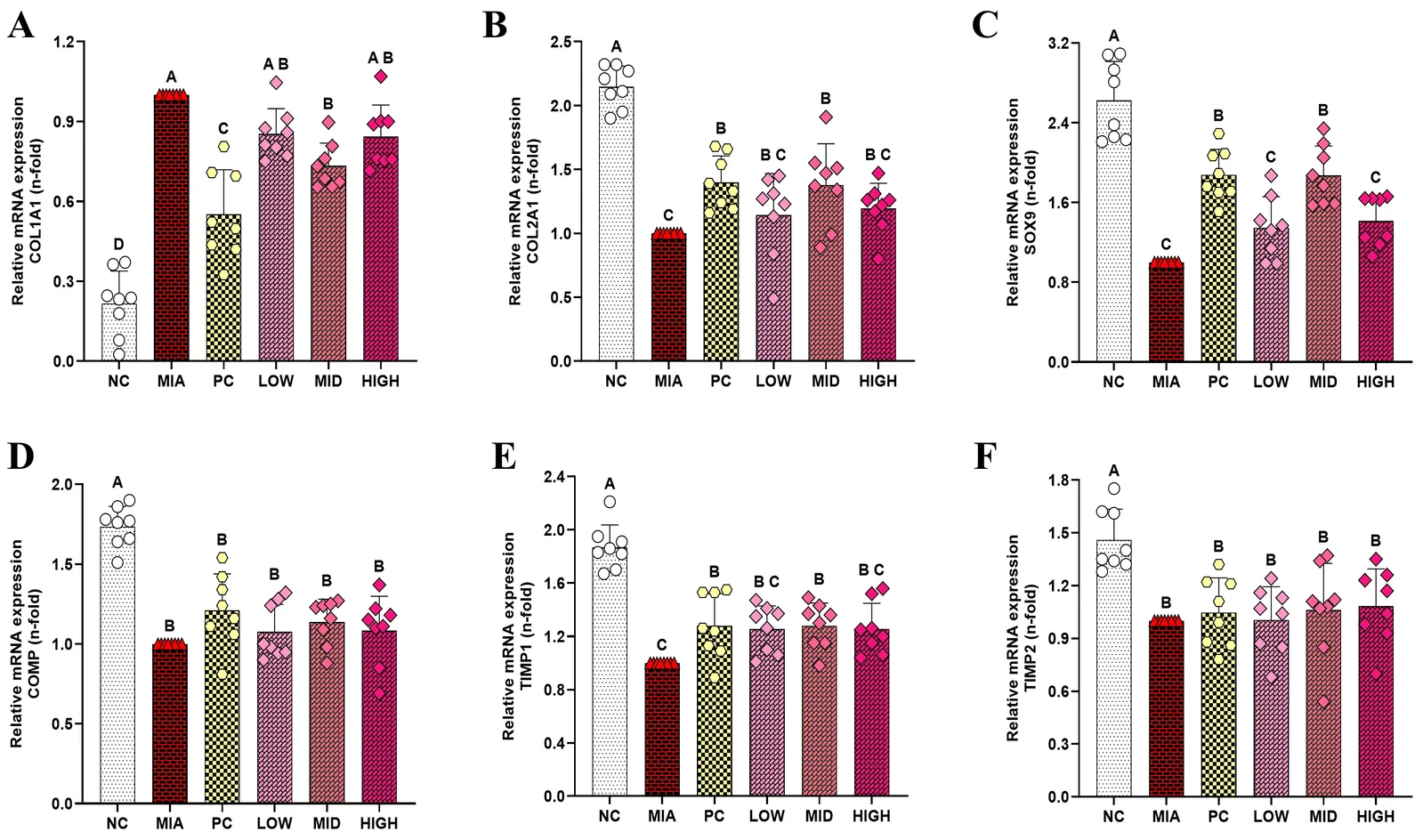
Effects of CHL on cartilage anabolic markers and ECM-related gene expression in OA rats. SD rats were randomly divided into six groups (*n* = 8/group): NC, MIA, MIA + celecoxib (PC, 10 mg/kg/day), and CHL-treated LOW (10 mg/kg/day), MID (30 mg/kg/day), and HIGH (300 mg/kg/day) groups. CHL and celecoxib were administered orally for 6 weeks, with OA induced after 2 weeks of pre-administration by intra-articular injection of MIA. The mRNA expression levels of (**A**–**F**) *Col1a1*, *Col2a1*, *Sox9*, *Comp*, *Timp1*, and *Timp2*, representing cartilage anabolic and ECM-related markers, were measured in joint cartilage tissues of OA rats using qRT-PCR. Values are presented as the mean ± SD (*n* = 8). Statistical significance is indicated by distinct letters, with different letters indicating statistically significant differences between groups. *Col1a1*, collagen type I alpha 1 chain; *Col2A1*, collagen type II alpha 1 chain; *Sox9*, SRY-box transcription factor 9; *Comp*, cartilage oligomeric matrix protein; *Timp*, tissue inhibitor of metalloproteinases.

To investigate the effects of CHL on cartilage anabolic markers and ECM-related gene expression, the mRNA expression levels of *Col1a1*, *Col2a1*, *Sox9*, *Comp*, *Timp1*, and *Timp2* were evaluated in the joint cartilage tissues of OA rats. MIA induction significantly increased *Col1a1* mRNA expression and decreased the expression of *Col2a1*, *Sox9*, *Comp*, *Timp1*, and *Timp2*, which was consistent with altered anabolic and ECM-related gene expression. However, oral administration of CHL, particularly at the MID-dose, significantly reduced *Col1a1* mRNA expression and increased the expression of *Col2a1*, *Sox9*, and *Timp1*. The PC group also showed reduced MIA-induced *Col1a1* expression and increased *Col2a1*, *Sox9*, and *Timp1* expression, with effects comparable to those observed in the CHL group at the MID-dose. Neither PC nor CHL treatment at any tested dose significantly affected the mRNA expression of *Comp* or *Timp2* (Figure 8A–F).

## 4. Discussion

OA is a progressive degenerative joint disease characterized by anatomical and physiological changes in joint-associated tissues, including cartilage degradation, subchondral bone remodeling, and osteophyte formation, ultimately resulting in impaired joint function. Although several pharmacological agents are commercially available for the management of OA, many primarily provide symptomatic pain relief and may be associated with adverse side effects following long-term use. Recently, natural compounds have received increasing attention as potential therapeutic alternatives because of their relatively low toxicity and diverse biological activities. Several studies have reported the protective effects of natural and plant-derived compounds in both in vitro and in vivo models of arthritis [35,36,37,38,39,40]. In this study, the effects of CHL were evaluated using an MIA-induced OA rat model.

Because ASU preparations may differ in their chemical compositions, comparisons among individual preparations should be interpreted with consideration of their specific formulations. Previous studies on ASU in MIA-induced OA evaluated different ASU preparations using structural, inflammatory, and pain-related outcomes [28,29]. In these studies, ASU administration was initiated after MIA induction, whereas CHL administration in the present study began 2 weeks before MIA injection and continued for the subsequent 4 weeks after OA induction. Thus, the present treatment schedule allowed for the assessment of the effects of CHL when administered before and during the development of MIA-induced OA, providing information on its effects across both the pre- and post-induction phases of the model. In addition, the present study evaluated a broader range of outcomes, including weight-bearing function, joint structural changes, serum biochemical markers, inflammatory and matrix-degrading mediators, and expression of cartilage-related genes. This integrated assessment provides a broader characterization of the effects of individual standardized ASU preparations on experimental OA.

Functional impairment and subchondral bone remodeling are the characteristic features of OA and are closely associated with cartilage degeneration and chronic inflammation. In the present study, MIA induction caused a marked reduction in weight-bearing capacity, indicating pain-associated joint dysfunction, consistent with previous findings in an MIA-induced OA model [32]. However, oral administration of CHL at all tested doses attenuated the MIA-induced reduction in weight-bearing distribution, consistent with improved weight-bearing function. Furthermore, micro-CT analysis and H&E staining of the knee joint revealed structural deformities, reduced meniscus volume, and subchondral bone alterations following MIA injection, consistent with MIA-induced joint structural alterations. Although the histopathological findings were evaluated qualitatively rather than using a validated scoring system, the observed morphological changes support the beneficial effects of CHL on joint structural integrity. Quantitative analysis of meniscal volume showed a decrease following MIA injection, whereas the PC- and CHL-treated groups showed an increase compared to the MIA-induced group; however, these protective effects were not statistically significant. Nevertheless, qualitative micro-CT findings and histological observations indicated that CHL treatment reduced MIA-induced structural abnormalities in the knee joint, suggesting a potential protective effect against MIA-induced joint structural changes. These findings are consistent with previously published reports [32,41].

Chronic low-grade inflammation is considered a major contributor to OA pathogenesis. Pro-inflammatory cytokines such as IL-1β, IL-6, and TNF-α stimulate NF-κB and MAPK signaling pathways, which further increase the expression of COX-2, NOS2, and PGE_2_, thereby exacerbating synovial inflammation and cartilage degeneration [6,7]. Furthermore, LTB4R, a high-affinity receptor for leukotriene B4, promotes the recruitment and activation of inflammatory cells [42], and enhances NF-κB activity [43]. In the present study, MIA induction in SD rats increased the mRNA expression levels of pro-inflammatory mediators, including *Il1b*, *Il6*, *Tnfa*, *Il12*, *Cox2*, *Nos2*, and *Ltb4r* in joint cartilage tissue, along with elevated serum levels of IL-1β, IL-6, TNF-α, and PGE_2_, closely reflecting OA-associated inflammatory responses. However, oral administration of CHL, particularly at the MID-dose, partially attenuated MIA-induced inflammation by reducing the mRNA expression levels of *Il6*, *Tnfa*, *Cox2*, *Nos2*, and *Ltb4r* in cartilage tissues, as well as serum levels of IL-6, TNF-α, and PGE_2_. These findings suggest that CHL may exert anti-inflammatory effects in MIA-induced OA, as reflected by the reduction in the expression of selected inflammatory mediators and inflammation-related genes.

MMPs mediate excessive degradation of ECM components during OA progression. MMP-1 and MMP-13 are involved in collagen degradation, whereas MMP-3 activates other MMPs to accelerate matrix degradation. Byun et al. reported the anti-arthritic effects of *Chrysanthemum zawadskii* var. *latilobum* in MIA-induced rats and IL-1β-induced human chondrocytes, highlighting the involvement of MMP-1, MMP-3, MMP-9, and MMP-13 [44]. Similarly, Lee et al. reported that *Curcuma longa* extract reduced MIA-induced MMP-2, MMP-3, MMP-9, and MMP-13 expression and exhibited anti-arthritic effects [41]. Consistent with these findings, MIA administration significantly increased the serum levels and mRNA expression of MMPs, including *Mmp1*, *Mmp2*, *Mmp3*, *Mmp9*, and *Mmp13*, consistent with increased matrix-degrading activity. However, CHL treatment at the MID dose markedly suppressed the mRNA expression levels of *Mmp1*, *Mmp3*, and *Mmp9* as well as the serum levels of MMP-1, MMP-2, MMP-9, and MMP-13, suggesting a potential protective effect against cartilage matrix degradation. Serum biomarkers associated with cartilage destruction, including CTX-II and COMP, were significantly reduced following CHL treatment, in agreement with the previously reported anti-arthritic effects of the natural compound isorhamnetin [45]. Taken together, these findings suggest that CHL may have protective effects against OA-associated cartilage degradation, as reflected by the reduction in selected matrix-degrading enzymes and cartilage degradation biomarkers.

Cartilage homeostasis is maintained through a dynamic balance between the catabolic and anabolic repair processes. *Sox9* is a key transcription factor involved in chondrocyte differentiation and the regulation of cartilage-associated genes, including *Col2a1* and aggrecan [46]. In the present study, MIA induction reduced the mRNA expression levels of *Sox9*, *Col2a1*, and the MMP inhibitors *Timp1* and *Timp2*, while increasing *Col1a1* expression in the cartilage tissue, consistent with altered cartilage anabolic and ECM-related gene expression. However, oral administration of CHL, particularly at the MID dose, attenuated these MIA-induced alterations by increasing the expression of anabolic markers, such as *Sox9* and *Col2a1*, while reducing MIA-induced *Col1a1* expression. In addition, CHL increased *Timp1* expression in the cartilage tissue, suggesting that CHL may influence cartilage matrix-related responses. These findings are consistent with those of previous reports demonstrating the chondroprotective and anabolic effects of *Spinacia oleracea* extract in an OA model [47].

Although the established phytosterol composition of CHL supports the presence of β-sitosterol, campesterol, and stigmasterol as the major phytosterols contributing to its total phytosterol content and potentially supporting its anti-arthritic effects, the non-targeted HR-LC/MS analysis provides complementary compositional information. Additional compounds tentatively annotated as (+)-discodermolide, epicoccamide, and brasilicardin C may or may not contribute to the anti-arthritic effects of CHL; however, their potential biological relevance remains to be established. Future studies using authentic reference standards and targeted analytical approaches are warranted to confirm these annotations and determine whether these compounds contribute to the observed anti-arthritic effects of CHL. These findings also highlight the importance of characterizing individual ASU formulations, as differences in source materials, manufacturing processes, and standardization may contribute to variations in commercial preparations. Importantly, the individual contributions of the tentatively annotated compounds to the observed biological effects of CHL could not be determined in the present analysis. Therefore, chemical profiling did not establish a direct relationship between any tentatively annotated individual compounds and the observed biological effects of CHL. Collectively, these findings provide a comprehensive characterization of the effects of specific CHL preparations used in this study. However, because no ASU preparation was included as a direct comparator, these findings should not be interpreted as evidence that CHL is more effective than ASU in general, or that it acts through mechanisms identical to those reported for other ASU preparations.

Although CHL treatment was associated with changes in inflammatory mediators, matrix-degrading enzymes, and cartilage-related genes, we did not directly investigate the exact molecular mechanisms underlying these effects. In particular, pathway-specific experiments were not performed to clearly determine whether CHL directly affects NF-κB, MAPK, or other inflammatory signaling pathways. Therefore, the observed changes in inflammatory and cartilage-related markers should be interpreted as associations rather than as evidence of direct pathway regulation. Further studies using pathway-specific protein analyses and functional mechanistic experiments are required to elucidate the molecular basis of the effects of CHL. In addition, CHL was administered 2 weeks before MIA induction and continued thereafter, resulting in a predominantly preventive experimental design that limited the therapeutic effects of CHL in OA. Further studies are needed to evaluate the effects of CHL in established OA models to confirm its therapeutic potential.

## 5. Conclusions

Collectively, these findings suggest that CHL attenuates OA-associated changes in MIA-induced OA in SD rats. CHL administration was associated with improved weight-bearing function, attenuation of joint structural alterations, reduction in selected inflammatory mediators and matrix-degrading enzymes, and changes in cartilage-associated anabolic and ECM-related markers. These findings suggest a broad biological response to CHL across functional, structural, biochemical, and cartilage-related outcomes in patients with experimental OA. However, the observed effects should not be interpreted as evidence of direct regulation of specific inflammatory or molecular signaling pathways, as mechanistic pathway-specific experiments were not performed in the present study. These effects were more consistently observed at the MID dose, whereas the LOW and HIGH doses showed comparatively weaker or nonsignificant responses across several markers, suggesting that the MID dose produced the most consistent effects under the conditions tested. However, an optimal therapeutic dose range was not established in the present study. Future studies incorporating broader dose ranges, pharmacokinetic/pharmacodynamic assessments, and dedicated dose-response analyses are warranted to clarify the dose-response characteristics and determine the optimal dose of CHL for OA management.

## Figures and Tables

**Table 1:** The list of primers used in the current study.

Gene Name	Forward (5′–3′)	Reverse (5′–3′)
*Col1a1*	ACATGTTCAGCTTTGTGGACC	TAGGGACCCTTAGGCCATTGT
*Col2a1*	TGAAGACCCAGACTGCCTCA	GCCCCTTTGGCCCTAATTTTC
*Sox9*	TCACCTACATGAACCCAGCG	AGCTGTGTGTAGACGGGTTG
*Comp*	CTCCAGGAGACTAATGCGGC	CGCAAGCGTCACATTCCATC
*Ltb4r*	GTCCTTGTGTACCGCACAGT	CCGTGATGGCTTCAAAGAGC
*Nos2*	TGGTGCAGAAGCACAAAGTCA	GGGAATAGCACCTGGGGTTTT
*Cox2*	GATGACGAGCGACTGTTCCA	TGGTAACCGCTCAGGTGTTG
*Il1b*	CCTATGTCTTGCCCGTGGAG	CACACACTAGCAGGTCGTCA
*Il6*	CACTTCACAAGTCGGAGGCT	TCTGACAGTGCATCATCGCT
*Il10*	GAAGGACCAGCTGGACAACA	GGGGCATCACTTCTACCAGG
*Il12*	ACCTCTGCCGAAGTCCAATG	ACATCTTAGGATCGGCCCCT
*Tnfa*	CAGCAGATGGGCTGTACCTT	AAATGGCAAATCGGCTGACG
*Mmp1*	CCCCAGCTACACATGGTACAC	GATGTGGTGTTGTTGCACCT
*Mmp2*	AAGGATGGAGGCACGATTGG	GGGAACTTGATGATGGGCGA
*Mmp3*	ATGCAGGGAAAGTGACCCAC	CCTCCATGAAAAGACTCAGAGGAA
*Mmp9*	TCTGCCTGCACCACTAAAGG	TCGGCTCGAGTAGGACAGAA
*Mmp13*	CTGGGCCCTGAATGGGTATG	CTCAAAGTGAACCGCAGCAC
*Timp1*	TCAGCCATCCCTTGCAAACT	AATCTGGATTCCGTGGCAGG
*Timp2*	CCCCTGTGGCCAATTGAAAAG	ACACTAGCGTGAACCCACTTG
*Actb*	GCAGATGTGGATCAGCAAGC	AGAAAGGGTGTAAAACGCAGC

Abbreviations: *Col1a1*, collagen type I alpha 1 chain; *Col2a1*, collagen type II alpha 1 chain; *Sox9*, SRY-Box transcription factor 9; *Comp*, cartilage oligomeric matrix protein; *Ltb4r*, leukotriene B4 receptor; *Nos2*, nitric oxide synthase 2; *Cox2*, cyclooxygenase 2; *Il*, interleukin; *Tnfa*, tumor necrosis factor-alpha; *Mmp*, matrix metalloproteinase; *Timp* 1/*Timp *2, tissue inhibitor of *Mmp* 1/2; *Actb*, β-actin.

**Table 2:** Tentative identification of compounds detected in CHL by HR-LC/MS in negative ionization mode.

Peaks	RT (min)	Putative Compounds	CAS No.	Area	Molecular Weight
1	1.937	Unknown	-	189 187 644	133.875 07
2	8.375	Unknown	-	134 212 487	136.978 24
3	8.746	Epicoccamide	606139-26-8	283 930 894	557.355 85
4	8.896	Saccharin	81-07-2	37 733 080	182.998 90
5	8.936	Leiwansterol A	-	44 705 017	460.354 54
6	8.997	Nahuoic acid E	-	107 268 111	552.365 61
7	9.032	Halymecin E	-	70 004 740	592.380 02
8	9.108	Brasilicardin C	-	265 149 854	569.355 97
9	9.980	N-Undecanoulglycine	83871-09-4	37 703 931	243.183 29
10	10.009	(+)-Discodermolide	127943-53-7	323 677 008	593.392 71

Abbreviations: CAS, Chemical Abstracts Service; CHL, standardized phytosterol-rich avocado/soybean unsaponifiable preparation; HR-LC/MS, high-resolution liquid chromatography-mass spectrometry; RT, retention time.

## Data Availability

The data supporting the findings of this study are available from the corresponding author upon reasonable request due to institutional and data-management considerations. Qualified researchers may request access to the underlying data from the corresponding author.

## Abbreviations

The following abbreviations are used in this manuscript:

ASU	avocado/soybean unsaponifiables
CHL	standardized phytosterol-rich avocado/soybean unsaponifiable preparation
COMP	cartilage oligomeric matrix protein
COX-2	cyclooxygenase-2
CT	Computed tomography
CTX-II	C-terminal cross-linked telopeptide of type II collagen
ECM	extracellular matrix
EPA	eicosapentaenoic acid
GAGs	glycosaminoglycans
HR-LC/MS	high-resolution liquid chromatography-mass spectrometry
IL	interleukin
MAPK	mitogen-activated protein kinase
MIA	monosodium iodoacetate
MMPs	matrix metalloproteinases
NF-κB	nuclear factor kappa-light-chain-enhancer of activated B cells
NO	nitric oxide
NOS2	inducible nitric oxide synthase
NSAIDs	nonsteroidal anti-inflammatory drugs
OA	osteoarthritis
OS	oxidative stress
PGE2	prostaglandin E2
ROS	reactive oxygen species
TIMPs	tissue inhibitors of metalloproteinases
TNF	tumor necrosis factor
ELISA	Enzyme-linked immunosorbent assay
